# Acquired uterine arteriovenous malformation in a patient with cornual pregnancy: A case report

**DOI:** 10.1097/MD.0000000000031629

**Published:** 2022-11-25

**Authors:** Yi Yan, Yong Jia, Belinda Lategan, Zarine Alexander, Alaa Awadalla, Ashraf Goubran

**Affiliations:** a Department of Radiology, Max Rady College of Medicine, Rady Faculty of Health Sciences, University of Manitoba, Winnipeg, MB, Canada; b Department of Pathology, Max Rady College of Medicine, Rady Faculty of Health Sciences, University of Manitoba, Winnipeg, MB, Canada; c International Medical Graduate Programs, Room 260 Brodie Centre 727 McDermot Avenue, University of Manitoba, Winnipeg, MB, Canada; d Obstetrics, Gynecology and Reproductive Sciences 260 Brodie Centre, 727 McDermot Avenue University of Manitoba, Bannatyne Campus Winnipeg, MB, Canada; e Ultrasound Department, Radiology & Diagnostic Imaging, St. Boniface General Hospital, Winnipeg, MB, Canada.

**Keywords:** cornual pregnancy, uAVM, ultrasound

## Abstract

Acquired uterine arteriovenous malformation (uAVM) is a rare disease and could occur after dilation and curettage, cesarean section, or neoplastic processes.

**Patient concerns:**

A 29-year-old female presented with acute right lower abdominal pain and positive beta human chorionic gonadotropin (β-hCG).

**Diagnosis:**

A 6 cm ectopic right cornual pregnancy was found on ultrasound examination.

**Interventions:**

She underwent a laparoscopic resection of the cornual ectopic pregnancy. She returned with extensive vaginal bleeding 6-month post surgery, and eventually diagnosed with arteriovenous malformation at the previous surgical site by Color Dopplor endovaginal ultrasound. Percutaneous transcatheter uterine artery embolization (UAE) was attempted, however, vaginal bleeding continued. She was taken to the operation room for a hysteroscopic ablation of uAVM.

**Outcomes:**

Complete cessation of the bleeding was achieved without hysterectomy.

**Conclusion:**

We report an extremely unusual case of acquired uAVM after a wedge resection of cornual pregnancy. Ultrasound evaluation of patients with post-operative persistent bleeding should be considered for evaluation of a possible arteriovenous malformation.

## 1. Introduction

A cornual pregnancy is a rare type of ectopic pregnancy that results from faulty implantation within the cornu of the uterus.^[1]^ A cornual pregnancy is life-threatening because of subsequent uterine rupture and uncontrolled hemorrhage that require an emergent hysterectomy.^[2]^

A uterine arteriovenous malformations (uAVM) is defined as an abnormal connection between arteries and veins that lack an intervening capillary network on histopathologic examination. AVMs in the female genital tract can be embryological or caused by pathologic pelvic conditions including inflammatory processes, neoplastic processes and uterine trauma.^[3]^ The most common cause of iatrogenic uterine trauma is surgical intervention such as cesarean section, myomectomy, or normal vaginal surgery.^[4]^ The most common presentation of arteriovenous malformations is menorrhagia. Massive hemorrhage is catastrophic if appropriate preoperative planning is not undertaken. Clinical examination can reveal a vascular thrill or pulsation in the vaginal fornices; however, the diagnosis often requires confirmatory imaging. Endovagninal ultrasonography with Doppler is the first modality commonly used for the assessment of uAVM.^[5]^ uAVMs can be also diagnosed with contrast-enhanced computed tomography (CT), and magnetic resonance.^[6]^ Angiography with arterial embolization can provide both diagnosis and treatment.^[7]^

We report an unusual case of a cornual pregnancy developed a subsequent uterine AVM after wedge resection, both of which were diagnosed by endovaginal ultrasound. It unfortunately failed uterine artery embolization (UAE) with massive recurrent vaginal bleeding, but finally managed with hysteroscopic ablation.

## 2. Case report

**Figure 1.**
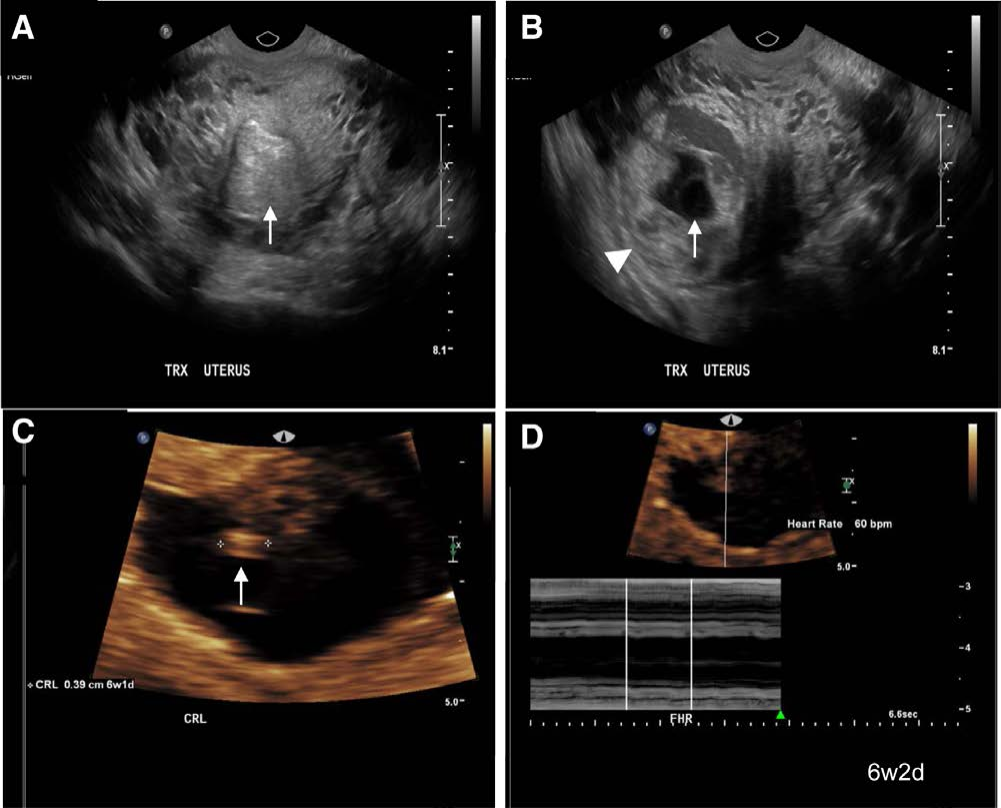
Ultrasound examination showed a live right cornual pregnancy. (A, B) Endometrial cavity is empty. An approximately 5 cm gestational sac is seen in the right cornual region as indicated with arrow. (C, D) A yolk sac seen and a fetal pole with a slow fetal heart rate of 60 beats per minute. Crown-rump length is 3.9 mm of estimated age 6 wks 1 d. Interstitial ectopic is implanted within the cornua surrounded by a thin myometrium (Arrow head).

**Figure 2.**
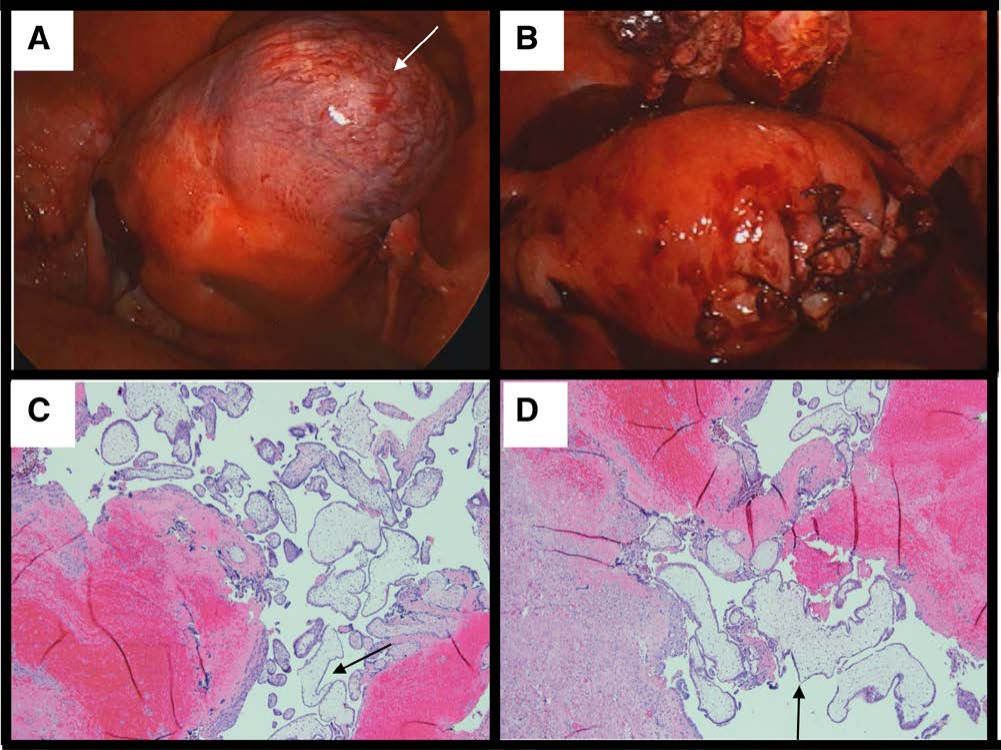
Intra-operative and pathological findings of cornual pregnancy. (A, B) Partial resection of the right uterine cornus was performed. (C, D) Photomicrograph with Haematoxylin-Eosin (H&E) stain shows villous tissue (arrow) invading myometrium. Around the implantation site, hemorrhage is also noted (H&E stain, ~20×).

A 29-year-old female with a remote history of a large 15 cm right ovarian dermoid cyst that necessitated laparoscopic right salpingo-oophorectomy. She presented initially with acute right lower pain with positive beta human chorionic gonadotropin (β-hCG) in March 2020. An endovaginal ultrasound demonstrated an empty endometrial cavity. However, an approximately 5 cm gestational sac was seen in the right cornual region surrounded by a thin myometrium as indicated in Figure 1A. Within the gestational sac (Fig. 1B), a yolk sac and a fetal pole with a slow fetal heart rate of 60 beats per minute were identified (Fig. 1C and D). Crown-rump length measured 3.9 mm of estimated gestational age 6 weeks 1 days. The empty uterine cavity with an eccentrically placed sac is consistent with an ectopic cornual pregnancy. The patient was counseled urgently by gynecologist who opted for medical treatment as surgical treatment carries a risk of uterine rupture and hysterectomy. She had subsequently received methotrexate. However, her β-hCG remained high, and the medical treatment was not successful. Hence, she underwent a laparoscopic excision of cornual pregnancy with a reconstruction of the right uterine cornu (Fig. 2A and B). The resected sample from right cornu shows chorionic villi and decidua in association with uterine smooth muscle, in keeping with cornual ectopic pregnancy. There is no evidence of gestational trophoblastic disease (Fig. 2C and D).

**Figure 3.**
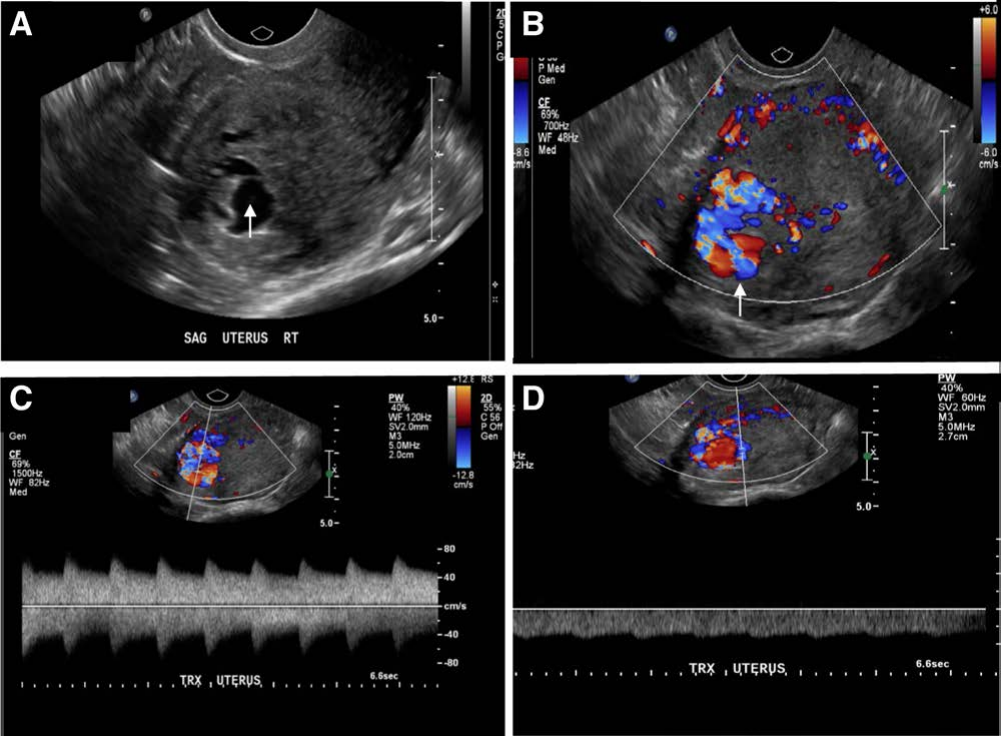
Ultrasound examination demonstrated an arteriovenous malformation (uAVM) at surgical bed. (A, B) There is a large cluster of vessels centered in the myometrium at the right fundal aspect of the uterus at the site of a previously cornual ectopic pregnancy. This occupies a volume of approximately 2.1 × 2.1 × 2.3 cm. (C, D) Pulse Doppler demonstrated a low resistance arterial as well as venous flow. An uAVM is suspected at the right fundal aspect of the uterus that site of previous cornual pregnancy.

**Figure 4.**
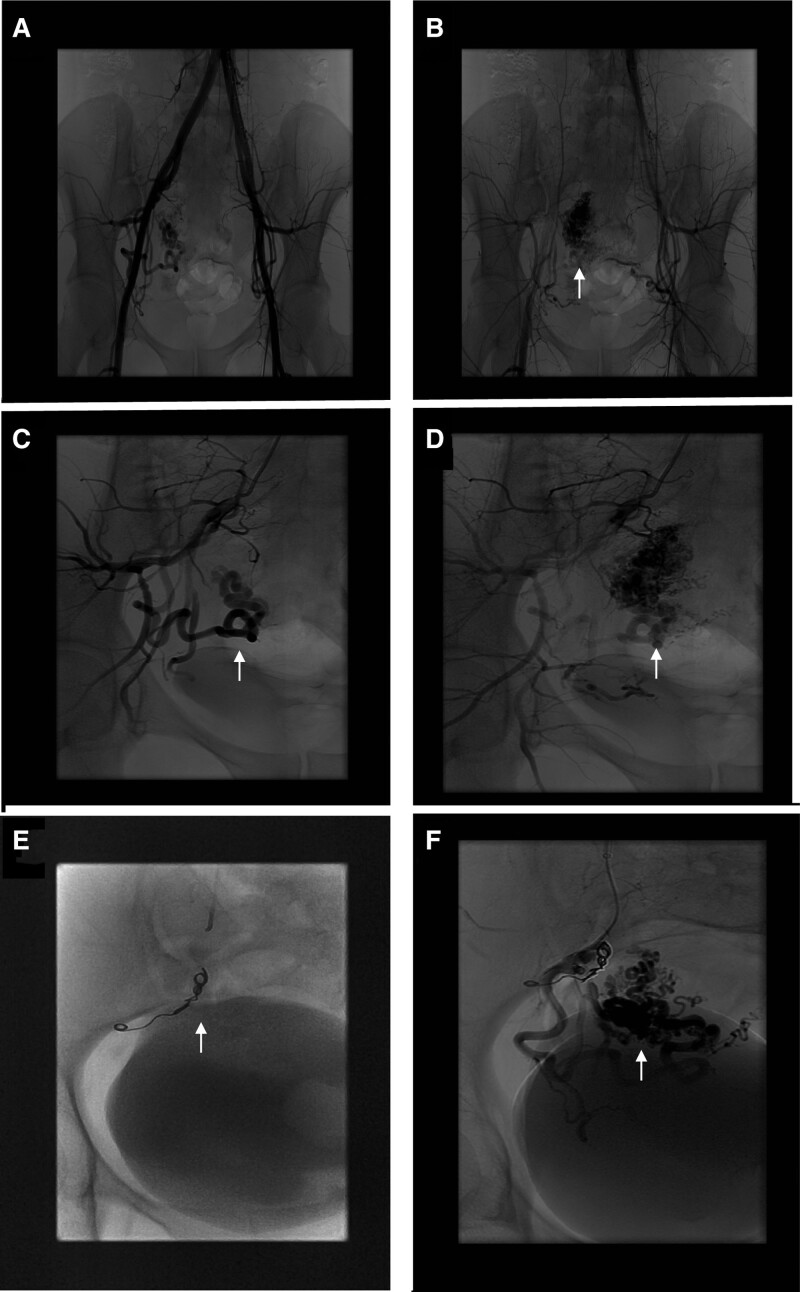
Conventional angiographic findings of uAVM. (A, B) The lower abdominal angiogram was performed followed by angiograms of both common iliac and internal iliac arteries. (C, D) Selective angiograms on the branches of the right internal iliac artery were performed followed by the left uterine artery. The malformation is demonstrated in the right upper quadrant of the uterus supplied by a large right uterine artery. In the region of the origin of the right uterine artery, the other branches of the internal iliac artery also originated. (E) There were significant difficulties in accessing the ostium of the right uterine artery. Coil embolization of at least 2 adjacent branches were performed to encourage cannulation of the right uterine artery. (F) The post-embolization arteriogram showed persistent contrast in uAVM denoting failure to occlude. uAVM = uterine arteriovenous malformation.

Four-month post surgery, the patient was presented at emergency department with severe vaginal bleeding. Her hemoglobin dropped from 127 down to 77. She received 2 units of packed blood cells and was also treated with tranexamic acid. An endovaginal ultrasound demonstrated a large cluster of vessels centered in the myometrium at the right fundal aspect of the uterus at the site of a previous cornual ectopic pregnancy. This occupies a volume of approximately 2.1 × 2.1 × 2.3 cm (Fig. 3A and B). Both low resistance arterial and venous flow was demonstrated (Fig. 3C and D). An arteriovenous malformation at the site of previous healing cornual pregnancy was therefore suggested and required an angiogram for confirmation. The angiogram revealed the presence of multiple feeding arteries mainly from the right uterine artery. An early draining vein was also observed at the fundus. The findings were consistent with an intrauterine arteriovenous malformation (Fig. 4A–D). An attempt was made to access the right uterine artery. However, there was difficulty in accessing the ostium of the right uterine artery. Coils did not manage to get into the arteriovenous shunt, hence the procedure was aborted. Coil embolization of at least 2 adjacent branches were performed to encourage cannulation of the right uterine artery (Fig. 4E). The post-embolization arteriogram showed persistent intrauterine arteriovenous malformation (Fig. 4F).

**Figure 5.**
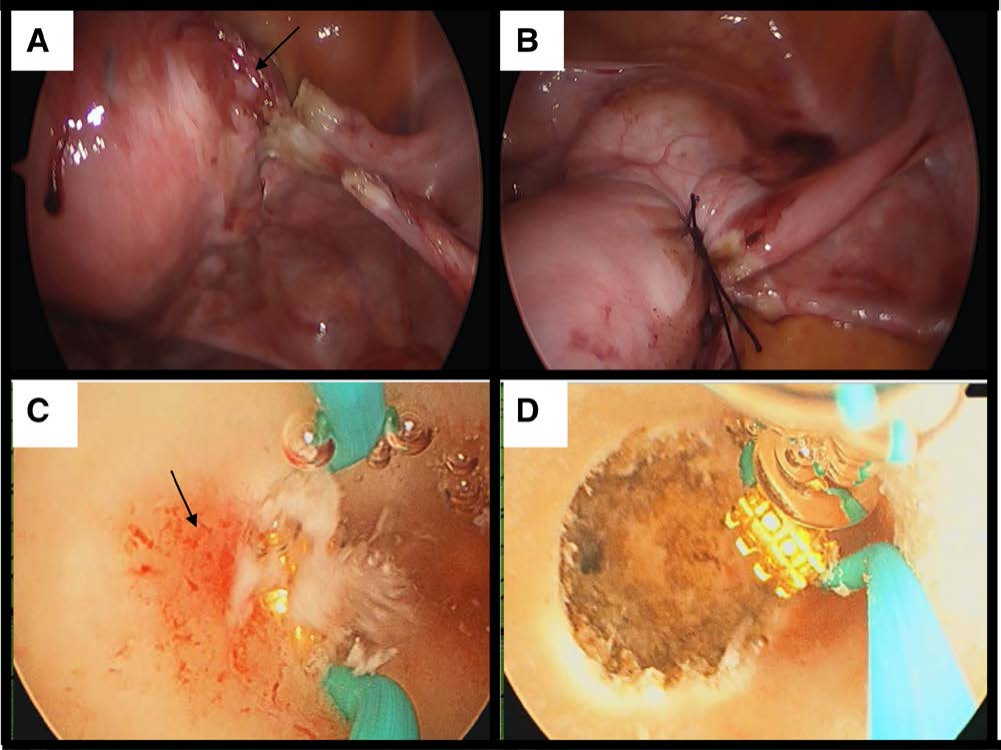
Emergency hysteroscopy with ablation of the arteriovenous malformation (A, B) Hystroscopic images of right uterine vessel showed a varicose-like structure, starting from the level of the internal OS reaching to the uterine cornu with significant pulsations. The vascular structure is likely corresponding the arteriovenous malformation that was described on imaging. Careful securing the malformation with suturing was performed and the extracorporeal knot tying technique allowed enough pressure to stop the blood supply into the abnormality. (C, D) The right uterine cornu had a bleeder that was giving arterial blood in spurts. A flat cylinder was used for coagulation with current of 80 watt. The bleeder was coagulated.

The patient continued to bleed vaginally. The discussion of an attempt to suture the arteriovenous shunt laparoscopically was initiated. Under the laparoscopy, an active bleeding was identified from a large tortuous vein and arterial supply above the ascending branch of the uterine artery (Fig. 5A). The uterine artery was isolated and secured with a figure-of-8 suture × 2 and that controlled the bleeding relatively well (Fig. 5B). Unfortunately, after 1 week, she came back to the hospital with emergency vaginal bleeding that dropped her hemoglobin from 85 down to 73. She was then consulted her for hysteroscopy, with the plan to ablate the inside of the healed area of the cornual ectopic pregnancy. She underwent an emergency hysteroscopy with ablation of the arteriovenous malformation bleeding spots in the uterus. Introoperatively, the right uterine cornu had a bleeder that was giving arterial blood in spurts (Fig. 5C). The bleeder was coagulated using flat cylinder (Fig. 5D). The endometrium around the bleeder was coagulated to get the deeper control on the bleeding point. The procedure was successful; the bleeding was finally settled. No hysterectomy is required up to today. The rest of the endometrium was left healthy; hence this patient can carry a pregnancy in future with no issues.

## 3. Discussion

To the best of our knowledge, the present case report is the first to describe a patient with an acquired uAVM due to a previous wedge resection for cornual pregnancy. This patient was considered as an acquired uAVM, because she undergone a wedge resection surgery, which likely is the cause of uAVM formation. The development of an uterine AVM after cornual ectopic pregnancy is extremely rare and potentially life-threatening. The optimal management of uAVM post cornual pregnancy has not yet been determined. Guan et al^[8]^ have previously reported the case of a large uAVM due to a missed cornual pregnancy with placenta accreta. More case reports of acquired AVM are found in patients with history of C-section scar ectopic pregnancy^[7,9]^ or fallopian tube pregnancy.^[10]^ These cases are similar to our case although no report has yet been published in patient with acquired AVM post wedge resection due to cornual pregnancy.

Cornual pregnancy, in other name interstitial pregnancy, is a rare type of ectopic pregnancy in which the embryo implants in the junction between the fallopian tube and the uterus.^[11]^ It occurs in about 4% of ectopic pregnancy but is often overlooked, diagnosed late and thus life-threatening.^[2,12]^ Diagnosis is quite challenging, because on ultrasound, the pregnancy often appears to be intrauterine engulfed by a thin myometrium.^[13,14]^ Sometimes only image clue is eccentric implantation of a gestational sac. The empty uterine cavity creates an echogenic interface leading to the eccentrically placed sac which is known as “interstitial line” sign.^[15]^

Conservative medical management with Methotrexate or surgical management such as wedge resection have been used to treat the cornual pregnancy.^[2,12,16,17]^ Traditionally, cornual pregnancies were treated with cornual resection or hysterectomy via laparotomy. Expectant management is an option for selected women with non-viable interstitial pregnancies and low serum β-hCG levels.^[12]^ In this situation, the β-hCG level was > 3000 mIU/mL with a live cornual ectopic pregnancy. After the failure of conservative treatment with Methotrexate, the laparoscopic cornual wedge resection was selected as further treatment plan in current case. No residual trophoblastic tissue was left in situ at the time of cornual wedge resection. The surgery however predisposed to potential formation of the acquired arteriovenous malformation. preexisting arteriovenous malformations is unlikely predate surgery, due to normal endovaginal imaging in early pregnancy. These vascular malformations have been proposed to be the result of angiogenesis promoted by cornual wedge resection. AVM was also large or superficial enough for the vessels to be exposed at the endometrium and subsequently caused catastrophic vaginal bleeding.^[18]^

Most uAVMs are diagnosed using less invasive methods such as ultrasonography, CT or MRI with a proper clinical setting.^[5,19]^ Transvaginal ultrasonography is often used for the initial evaluation of uAVM.^[5]^ In gray scale images, uAVMs appear as irregular cystic spaces which can vary in size. Careful assessment with Color and pulse Doppler is crucial since the gray scale findings are entirely nonspecific. Color Doppler usually shows tortuous vascular structures, with a multidirectional flow and apparent reverse flow due to aliasing. Pulse Doppler analysis shows low resistance flow with high velocity within the lesion.^[5]^ Accurate systolic velocities could not be obtained due to challenges with angle correction. Pelvic MRI assessment is often not required, but useful to identify the extent of the lesion. If performed, uAVM typically manifests as multiple serpentine flow voids on both T1 and T2-weighted sequences.^[6]^ Angiography is the gold standard for the diagnosis of AVMs but it is rarely performed for diagnosis alone due to its invasive nature.^[7]^ Both clinical history and imaging findings should be taken into consideration for radiologists and clinicians when making diagnosis of uAVM. Having a high index of suspicion for AVM among the differential diagnosis is critical, as it can lead to catastrophic hemorrhage and life threatening. Histologically, uterine AVMs would show dilated arteries and veins with different size and malformed, varied thickness of wall. The overlying endothelial cells are bland, lacking cytological atypia or mitosis.^[20]^

Although small uterine AVM tend to resolve spontaneously, most of uterine AVM would require either conservative medical management or surgical innervations including UAE, laparoscopic or hysteroscopic ablation.^[3]^ UAE has recently proved to be an effective alternative mode of treatment which is less invasive than surgery and has the benefit of fertility preservation.^[7,9,21]^ In a rare situation, uAVM ablation would be required under laparoscopy or hysteroscopy. The last resort for uAVM treatment is a total hysterectomy.^[8,19]^

In our case, the conventional uterine artery angiogram was initially performed for the purpose of treatment plan, and attempted percutaneous transcatheter UAE was performed. However, the intra-operative UAE course was challenging. Subsequent laparoscopic uAVM ablation was also insufficient due to the complexity of the patient’s vascular pattern that most of the dilated vessels are located adjacent to the endometrium and might have instigated surrounding neovascularization. For the above reasons, UAE and laparoscopic ablation have only temporarily alleviated this patient’s symptoms, and uAVM continue to bleed. The decision was made to proceed with a hysteroscopic ablation. The procedure was successful; the bleeding was finally settled. There would still be a role for surgical management such as a total hysterectomy in cases where hysteroscopic ablation has failed.

## 4. Conclusion

We report an unusual case of acquired uAVM after wedge resection of cornual pregnancy. The diagnostic dilemma requiring a multi-disciplinary approach involving Gynecologists, Sonologists and Interventional Radiologists. Ultrasound evaluation of patients with post-operative persistent bleeding should be considered for evaluation of a possible arteriovenous malformation.

## Author contributions

**Conceptualization:** Yi Yan, Belinda Lategan.

**Data curation:** Yi Yan.

**Supervision:** Yi Yan, Ashraf Goubran.

**Writing – original draft:** Yi Yan, Yong Jia, Zarine Alexander, Alaa Awadalla, Ashraf Goubran.

**Writing – review & editing:** Yi Yan, Yong Jia, Ashraf Goubran.
